# Mr. Morris and Lord Knutsford

**Published:** 1918-10-26

**Authors:** W. A. Chapple

**Affiliations:** House of Commons


### THE EDITOR'S LETTER-BOX.

**[Correspondence on all subjects is invited, but we cannot in any way be responsible for the opinions expressed by our correspondents, who must give their name and address as a guarantee of good faith, but not necessarily for publication. Correspondents are reminded that brevity of style and conciseness of statement greatly facilitate early insertion.]**

### THE LONDON HOSPITAL'S NURSES.

### Mr. Morris and Lord Knutsford.

*To the Editor of* THE HOSPITAL.

SIR,—If Mr. Morris gave Lord Knutsford away in a previous issue of THE HOSPITAL by admitting that the "London" took nurses from their studies and training in the wards at the end of their second year and farmed them out for profit. by paying them 13s. 5d. per week while they drew £2 12s. 6d. for their services and pocketed the difference, how much more have the "probationer nurses" out-Heroded Herod in your last issue by their scathing indictment of the London Hospital.

Lord Knutsford declares in his letter to you on the same page as appears this *genuine*—(put it in italics, please)—this *genuine* letter from oppressed nurses, "I have cared for them so much!" Yet on the same page in your issue your correspondents give their pathetic testimony, and you yourself, Sir, are "assured" that it is "correct." They say that the London Hospital probationers dare not complain "simply from fear"; that "feeling is running very high just now at the 'London' . . ."; that "all of us would be only too pleased to have a three-years' training . . . and we feel we are still untrained at the end of two years."

How strange the contrast between these probationer nurses and the other ? . . . . . apologists for the London Hospital, who have bespattered your pages with a camouflage of irrelevancies and who show much more anxiety to curry favour with their employers than to render their sisters a service.

If Lord Knutsford would come down from his lofty autocracy and try to learn from other London hospitals how to train and treat nurses for the benefit of the nursing profession and the sick whom they are destined to attend, he might get from others, or at least merit, a portion of the credit he gives to himself.—Yours faithfully, W. A. CHAPPLE.

House of Commons, October 19, 1918.

